# Co‐targeting driver pathways in prostate cancer: two birds with one stone

**DOI:** 10.15252/emmm.201808928

**Published:** 2018-03-25

**Authors:** Amina Zoubeidi, Martin E Gleave

**Affiliations:** ^1^ The Vancouver Prostate Centre Department of Urologic Sciences University of British Columbia Vancouver BC Canada

**Keywords:** Cancer, Pharmacology & Drug Discovery

## Abstract

Co‐targeting strategies strive to improve cancer outcomes by combining therapies under contextualized genetic and environmental conditions that selectively target exploitable alterations in tumor cells. Adaptive survival pathways triggered by inhibition of driver genes in the androgen receptor (AR) or PI3K/AKT pathways are of great interest, since they are among the most frequently altered in castrate‐resistant prostate cancer (CRPC). Unfortunately, negative feedback loops exist between the AR and PI3K/AKT pathways such that targeting AR leads to activation of PI3K/AKT signaling, while PI3K/AKT pathway inhibition leads to increased AR transcriptional activity. Hence, targeting both pathways provides an opportunity for conditional lethality and a high therapeutic index. In this issue of *EMBO Molecular Medicine*, Yan *et al* (2018) present an elegant study showing that histone deacetylase 3 (HDAC3) acts as a common upstream activator of both AR and AKT signaling pathways, and use HDAC3 inhibitors as a monotherapy to co‐target two major pathways driving CRPC growth.

The discovery that castration‐resistant prostate cancer (CRPC) most often remains fueled by androgen receptor (AR) signaling ushered in the development and clinical integration of highly potent AR pathway inhibitors. These agents have undoubtedly benefited patients with CRPC, but are merely palliative as resistance inevitably emerges. There is a need to develop more active regimens, either by combining currently approved drugs, or via design of novel biologically rational regimens to create contextual lethality based on genomic biomarker enrichment, and/or co‐targeting adaptive survival pathways activated by AR pathway inhibitors. Indeed, impressive results from CHAARTED (NCT00309985), STAMPEDE (NCT00268476), and LATITUDE (NCT01715285) clinical trials highlight significant benefits of combination versus monotherapy regimens, as well as early versus late intervention in advanced prostate cancer. These clinical trials combined androgen‐deprived therapy (ADT) with chemotherapy (for CHAARTED, STAMPEDE), and ADT with abiraterone (for LATITUDE, STAMPEDE) in advanced castrate‐sensitive prostate cancer patients. The studies all report a remarkably similar 40% reduction in death rate and provide precedent for future effective combination regimens across the prostate cancer treatment landscape.

Castrate‐resistant prostate cancer most often progress with an activated AR despite potent AR pathway suppression, supported by a vast network of pro‐survival genes and growth factor pathways, including the PI3K/AKT pathway. Indeed, the AR (via amplification, mutation, variants) and PI3K/AKT (via Pten loss) pathways are the two most frequently genomically altered pathways in CRPC. While clinical responses are common with AR pathway inhibitors, responses using PI3K inhibitors are rare in preclinical models or patients with CRPC. Monotherapy with AR or AKT inhibitors results in reciprocal crosstalk activation that supports emergence of acquired resistance (Carver *et al*, 2011; Mulholland *et al*, 2011). Genomically, 70% of prostate cancers harbor PTEN alterations and 10% harbor SPOP mutations. Both phenotypes are associated with activated AR and PI3K/AKT pathways despite the fact that, in early treatment‐naïve localized disease, *SPOP* mutations are mutually exclusive with genomic alterations in PI3K/AKT pathway. Interestingly, these alterations can co‐occur in CRPC. Expression of mutant SPOP activates PI3K/AKT pathway and upregulates AR signaling, maintaining AR transcriptional activity against PI3K/AKT‐mediated negative feedback, effectively activating the two most common driver pathways critical in prostate cancer. Hence, combined blockade of these pathways may delay treatment resistance and significantly improve patient outcome.

The study by Carver *et al* (2011) was the first to demonstrate that the AR and PI3K pathways co‐regulate one another via reciprocal negative feedback, such that inhibition of one activates the other. Mechanistically, inhibition of the PI3K pathway increased AR signaling in PTEN‐deficient prostate cancer in part via relief of negative feedback to HER kinases; conversely, AR antagonism relieves feedback inhibition of AKT by reducing FKBP5‐mediated stability of the phosphatase PHLPP. While tumor cells can adapt and survive when either single pathway is inhibited, combined inhibition of PI3K/AKT and AR signaling using the PI3K/mTOR inhibitor BEZ235 and the AR antagonist enzalutamide (ENZ) significantly delayed castrate‐resistant LNCaP tumor progression. Similar data were reported by Thomas *et al* (2013) and Toren *et al* (2015); increased AR transcriptional activity observed using monotherapy with the AKT inhibitor AZD5363 was overcome by combining AZD5363 with ENZ, resulting in synergistic inhibition of cell proliferation and induction of apoptosis, and delayed CRPC tumor growth *in vivo*. These data provided preclinical proof of principle to support evaluation of combination AZD5363 and ENZ in the clinic (NCT02525068). Another approach to co‐targeting AR and AKT pathways focussed on stress chaperone proteins activated by AR pathway inhibitors, coordinated by a feed‐forward loop involving p90rsk‐mediated phospho‐activation of YB‐1 with subsequent induction of CLU and AKT/MAPK activity. Co‐targeting the AR (with ENZ) and molecular chaperone CLU (with OGX‐011) repressed ENZ‐induced activation of AKT and MAPK pathways, accelerated AR degradation and repressed AR transcriptional activity through mechanisms involving decreased YB‐1‐regulated expression of the AR co‐chaperone, FKBP52, which synergistically delayed CRPC LNCaP tumor progression *in vivo* (Matsumoto *et al*, 2013).

The data presented by Yan *et al* (2018) describe an elegant approach to co‐target these pathways by inhibiting histone deacetylase 3 (HDAC3), an upstream regulator for both AR and AKT pathways, effectively eliminating two birds with one stone (Fig 1). HDAC3, which is upregulated in prostate cancer (Weichert *et al*, 2008), facilitates lysine‐63‐chain polyubiquitination and phosphorylation of AKT in prostate cancer cells, a non‐nuclear effect mediated by AKT deacetylation at lysine 14 and 20 residues and HDAC3 interaction with the scaffold protein APPL1. Targeted inhibition of HDAC3 blocks interaction with APPL1, decreasing AKT acetylation and thereby inhibiting AKT phosphorylation. In addition, HDAC inhibition decreases AR full‐length and splice variant mRNA levels in the PTEN loss and SPOP mutant prostate cancer models. HDAC3 inhibitor shows efficacy in these two prostate cancer subtypes that share activated AR and AKT pathways. This preclinical proof of principle is encouraging and will help guide bench‐to‐bedside translation to integrate HDAC3 inhibitor into clinical trials. Key issues include evaluation as monotherapy or as part of a combination regimen with an approved AR pathway inhibitor, as well as biomarker enrichment (e.g., PTEN and/or SPOP mutated, AR amplified) to include those more likely to respond. Targeting AKT and AR was more profound when combined with castration (ADT) in prostate cancer (Toren *et al*, 2015). Beyond the focus on AR and AKT pathways, HDAC3 inhibition may also enhance activity of PARP inhibitors (Ha *et al*, 2014) or chemotherapy (Long *et al*, 2017), supporting potential combinations in DNA repair‐deficient cancers, or when docetaxel is indicated.

**Figure 1:**
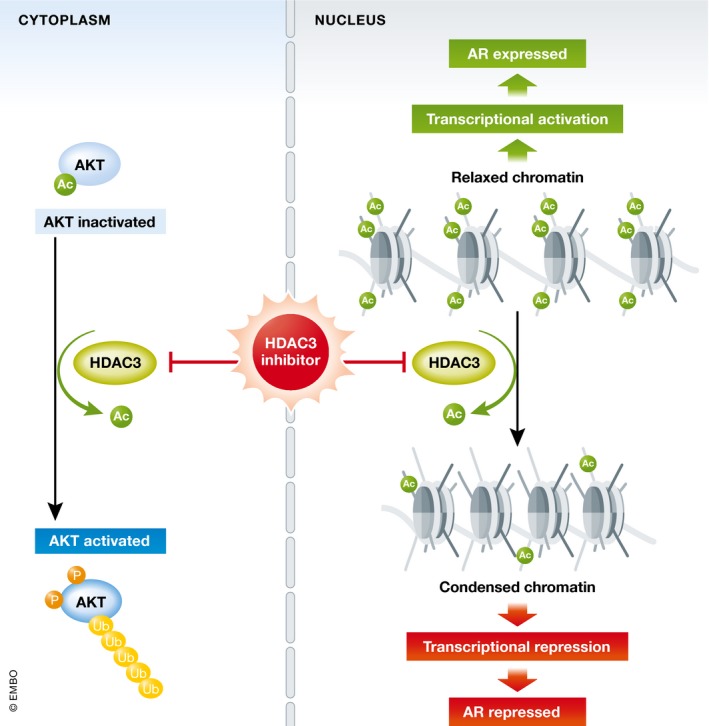
HDAC3 inhibitor effects on AKT and AR pathways HDAC3 integrates major signaling pathways in prostate cancer: the androgen receptor and the AKT pathways. Targeting HDAC3 using a small‐molecule inhibitor or siRNA inhibits AKT phosphorylation and HDAC3 binding to APPL1 in the cytoplasm, while it represses AR transcription via histone deacetylation and condensed chromatin in the nucleus.

In summary, the data presented by Yan *et al* (2018) define a mechanism‐based targeting of HDAC3 upstream of two key genomic altered pathways in prostate cancer and provide preclinical proof of principle to guide inhibitor development toward the clinic.
